# Scanning of paroxysmal atrial fibrillation as an etiological risk factor in patients with acute ischemic stroke: prospective study

**DOI:** 10.1590/1516-3180.2021.0156.R2.08062021

**Published:** 2022-02-21

**Authors:** Zahide Betül Gündüz, Ahmet Lutfi Sertdemir, Zafer Buyukterzi

**Affiliations:** I MD, PhD. Assistant Professor, Department of Neurology, Saglik Bilimleri University, Konya State Hospital, Konya, Turkey.; II MD, PhD. Assistant Professor, Department of Cardiology, Necmettin Erbakan University, Konya, Turkey.; III MD, PhD. Associate Professor, Department of Cardiology, Saglik Bilimleri University, Konya State Hospital, Konya, Turkey.

**Keywords:** Ischemic stroke, Electrocardiography, ambulatory, Stroke, acute, Paroxysmal atrial fibrillation, Holter electrocardiography, Cryptogenic stroke

## Abstract

**BACKGROUND:**

Prevention of recurrence of stroke depends on recognition of the underlying mechanism of ischemia.

**OBJECTIVE:**

To screen patients who were hospitalized with diagnosis of acute ischemic stroke in terms of atrial fibrillation (AF) with repeated Holter electrocardiography recordings.

**DESIGN AND SETTING:**

Prospective study conducted at Konya Education and Research Hospital, Turkey.

**METHODS:**

Patients with a diagnosis of acute ischemic stroke, without atrial fibrillation on electrocardiography (ECG), were evaluated. Their age, gender, histories of previous ischemic attack, occurrences of paroxysmal atrial fibrillation (PAF) and other risks were assessed during the first week after acute ischemic stroke and one month thereafter. ECG recordings were obtained from 130 patients through 24-hour ambulatory Holter. Patients without PAF attack during the first Holter were re-evaluated.

**RESULTS:**

PAF was detected through the first Holter in 33 (25.4%) out of 130 acute ischemic stroke patients. A second Holter was planned for 97 patients: 53 (54.6%) of them could not attend due to COVID-19 pandemic; while 44 (45.3%) patients had the second Holter and, among these, 4 (9.1%) had PAF. The only parameter associated with PAF was older age. Four (10.8%) of the 37 patients with PAF had also symptomatic carotid stenosis.

**CONCLUSIONS:**

Detecting the presence of PAF by screening patients with no AF in the ECG through Holter ECG examinations is valuable in terms of changing the course of the treatment. It should be kept in mind that the possibility of accompanying PAF cannot be ruled out in the presence of other factors that pose a risk of stroke.

## INTRODUCTION

Atrial fibrillation (AF) consists of atrial arrhythmia characterized by loss of P waves on electrocardiography, with one or more attacks for at least 30 seconds. Although AF is more common in the elderly or individuals with other cardiovascular risk factors, it is the most common type of arrhythmia, affecting approximately 3% of the adult population. Symptoms associated with AF can be observed in all of its subtypes, seen as paroxysmal, persistent or permanent AF. However, some patients with AF associated with stroke during or after stroke are also asymptomatic. It is known that AF increases the risk of ischemic stroke four to fivefold.^1^

It is important to reveal the etiology of stroke and to reduce the risk of stroke recurrence through putting appropriate treatment options into effect. However, in approximately a quarter of all ischemic strokes, the underlying factor cannot be revealed.^2^ For example, in the TOAST classification, this group is called cryptogenic stroke.

With developments in the field of neuroradiology and cardiological examination, and widespread access to these examinations, the definition of cryptogenic stroke has been questioned as a result of clarification of the etiology. This has been achieved through advanced examination methods among some of the patients who had previously been diagnosed as presenting cryptogenic stroke. Thus, the term ‘Embolic Stroke of Undetermined Source’ (ESUS) has been introduced.^3^

ESUS is held responsible for 20% of ischemic strokes.^4^ By definition, ESUS consists of a non-lacunar brain infarction with no demonstrable proximal arterial stenosis or cardioembolic source, and with a clear indication for anticoagulation.^1^ Studies have focused on the idea that a large proportion of ESUS patients may have silent paroxysmal AF (PAF).^5^

In the 2020 guidelines of the European Society of Cardiology (ESC) for patients with acute ischemic stroke or transient ischemic stroke (TIA) without known AF, it is recommended that after a short electrocardiography (ECG) recording in the first 24 hours, continuous ECG monitoring should be implemented for at least 72 hours, if possible (class 1, level B). It is recommended that AF should be scanned through long-term non-invasive ECG monitors or implantable cardiac monitors in selected patients with no known AF (class 2a, level B).^6^

However, not all stroke patients benefit from long-term ECG monitoring. Long-term ECG recording should be selected for patients who are considered to be at risk of developing AF (e.g. elderly individuals with cardiovascular risk factors or patients with presence of comorbidities, high left atrium remodeling index and high C2HEST score), patients whose condition is suggestive of embolic stroke and patients with cryptogenic stroke.^6^

Although it is recommended in the guidelines that Holter monitoring should be implemented 72 hours after stroke, it is obvious that each center should do its own planning according to the possibilities available. It needs to be borne in mind that some treatment centers do not have Holter monitoring opportunities; and that in centers that do have Holter monitoring, the devices are often limited to 24-hour recording.

## OBJECTIVE

In our center, where 72-hour Holter monitoring is not available, we planned our study based on the idea that recurrent 24-hour Holter recordings could increase the chance of picking up AF attacks that could not be detected in the first Holter. Moreover, we investigated whether Holter monitoring would produce different results in stroke patients whose stroke etiology could not be elucidated or correlated with other reasons.

## METHODS

The protocol for this prospective study was approved by the Ethics Committee of Selcuk University Medical Faculty (protocol number: 2019/347; date: November 27, 2019) and was funded by the Medical Specialty Education Board of the Saglik Bilimleri University, Konya Education and Research Hospital (protocol number: 48929119/774; date: May 2, 2020), in Turkey.

In our study, patients who were hospitalized in the Department of Neurology, Saglik Bilimleri University, Konya Education and Research Hospital, with a diagnosis of acute ischemic stroke, who did not show AF on ECG and who had not previously been diagnosed with PAF, were included. We sought to include patients who were euthyroid. In addition, we determined that the patients should not have valvular heart disease, which would be an indication for anticoagulant therapy on echocardiography (ECHO). The results from 24-hour Holter monitoring examinations completed in the first week, at the acute ischemic stroke clinic and at the end of the first month, among patients who we recruited over a six-month period, were evaluated according to age, gender, histories of previous ischemic attack and other risk factors.

Holter ECG monitoring was planned and implemented for patients during their hospitalization in the first week (days 0-7) and at the end of the first month (days 30-45), during which routine outpatient clinic control was planned after discharge, twice for 24 hours. No control Holter was required for patients who presented PAF in the first Holter.

Patients for whom it was planned to continue their post-discharge check-ups at an external center and patients whose general condition was bad enough to require monitoring in intensive care were not included in the study group. The examination and treatment plans for any patients who were hospitalized in the neurology clinic with a diagnosis of acute ischemic stroke but did not want to be included in the study were arranged by the neurologist who followed these patients.

Twenty-four hour ambulatory Holter ECG recordings were obtained from all the 130 patients who agreed to participate in the study and whose conditions were in accordance with what was desired for this study (Risingmed cv3L Holter system, Beijing, China). Rapid irregular atrial activity, characterized by absence of P waves that was observed for longer than 30 seconds in 24-hour Holter recordings, was reported as PAF.

Holter ECG appointments were given to patients who did not have a PAF attack in the first Holter, for dates that complied with the outpatient clinic controls one month later, at discharge. Control Holter results were collected and the data were transferred to a computer environment.

The data were analyzed using the SPSS 22.0 program (SPSS Inc., Chicago, Illinois, United States). Frequency and percentage values were used for categorical (nominal and ordinal) data. For numerical data, minimum and maximum values were given, along with the median value. In comparisons of categorical data, Fisher’s exact test was used when it met the assumptions required for the test, and when those of the chi-square test could not be met. In the analyses on all hypothesis tests, the significance level (P-value) was taken as 0.05.

## RESULTS

The ages of the 130 patients included in the study ranged from 31 to 92 years (interquartile range, IQR 62-78), with a median value of 69.5 years. The sample consisted of 70 men (53.8%) and 60 women (46.2%). There was no significant relationship between the sexes and the presence of PAF.

While 106 of the patients (81.5%) were evaluated after their first ischemic stroke attack, 19 (14.6%) were evaluated after their second and five (3.8%) after their third attack. All 11 patients (8.5%) who were evaluated as presenting transient ischemic attack were at their first attack. There was no statistically significant association between recurrent stroke attacks and the presence of PAF.

Regarding concomitant diseases, 87 patients (66.9%) had essential hypertension, 47 (36.2%) had diabetes mellitus, 28 (21.5%) had coronary artery disease and six (4.6%) had congestive heart failure. No statistically significant association was found between any of these diseases and the presence of PAF.

The number of patients using antiaggregant (acetylsalicylic acid, 100-300 mg) for various reasons was 35 (26.9%). No relationship could be established between presence of PAF and occurrence of ischemic stroke despite antiaggregant treatment. The patients’ characteristics are presented in Table 1.

**Table 1.** Demographic characteristics

	n (%)
**Male/female**	70/60
**Median age in years (IQR)**	69.5 (IQR 62-78)
**Evaluation time**	
First stroke attack	106 (81.5%)
Second stroke attack	19 (14.6%)
Third stroke attack	5 (3.8%)
**Concomitant diseases**	
Essential hypertension	87 (66.9%)
Diabetes mellitus	47 (36.2%)
Coronary artery disease	28 (21.5%)
Congestive heart failure	6 (4.6%)
**Using antiaggregant**	35 (26.9%)

IQR = interquartile range.

Twenty (16%) of the patients were over 80 years old. While PAF was detected in 24 (21.8%) of the 110 patients who were under 80 years of age, nine (45%) of the 20 patients aged 80 and over had PAF. There was a statistically significant difference (P = 0.028) between these age groups, such that the presence of PAF was found to be associated with increasing age (Table 2).

**Table 2.** Evaluation of PAF in the first Holter, according to the age group of the patients with stroke

	PAF	
	Positive	Negative
< 80 years old (n = 110)	24 (21.8%)	86 (78.2%)
≥ 80 years old (n = 20)	9 (45.0%)	11 (55.0%)

P-value: 0.028. PAF = paroxysmal atrial fibrillation.

The median value of the modified Rankin score (mRs) was 3 (IQR 1-3); 62 patients (47.7%) were independent (mRs 0-2) and 67 (51.5%) were dependent (mRs 3-5). One patient (0.8%) died due to non-neurological causes (mRs 6). There was no significant relationship between mRs and the presence of PAF.

The median value on the National Institutes of Health Stroke Scale (NIHSS) was 6 (IQR 4-9). No significant difference was found between a group with NIHSS 0-4 and a group with NIHSS 5 and above, in terms of PAF relationship.

The median value for CHA2DS2-VASc was 5 (IQR 4-6). No statistical relationship between CHA2DS2-VASc score and PAF was revealed.

PAF was detected in the first Holter in 33 (25.4%) of the 130 patients included in the study. Control Holter appointments for 53 (54.6%) out of the 97 patients who were not found to have PAF in the first Holter were canceled during the period when elective examinations were delayed in our hospital due to the COVID-19 pandemic. The other 44 patients (45.3%) were scanned through a second Holter, and four (9.1%) of these patients had a PAF attack during this control (Table 3).

**Table 3.** PAF results from first and control Holters

	Number of patients		PAF	
	Planned	Performed	Positive	Negative
First Holter	130	130	33 (25.4%)	97 (74.6%)
Second Holter	97	44	4 (9.1%)	40 (90.9%)

PAF = paroxysmal atrial fibrillation.

The first choice for carotid-vertebral artery imaging was as follows: Doppler ultrasonography for 116 (89.2%) of the patients, computed tomography (CT) angiography for nine (6.9%) and magnetic resonance imaging (MRI) angiography for five (3.85).

As the first examination or a further examination, 19 patients underwent CT angiography and 24 patients underwent MRI angiography. For seven patients who underwent CT angiography and six patients who underwent MRI angiography, evaluation of advanced stenosis was planned, to be performed using digital subtraction angiography (DSA). For four patients with advanced stenosis on CT angiography or MRI angiography, presence of PAF in the first Holter was also revealed.

Thrombolytic therapy and/or thrombectomy were applied to all patients who were evaluated in the hyperacute period and for whom these were indicated. In 14 patients, only intravenous thrombolytic therapy was applied, in three patients only thrombectomy was applied and in four patients intravenous thrombolytic therapy and thrombectomy were applied. There was no significant relationship between the patients who underwent thrombolytic therapy and/or thrombectomy and the presence of PAF.

## DISCUSSION

If persistent AF is detected through routine ECG evaluation during stroke, it is easy to demonstrate its relationship with stroke. However, it may not always be easy to detect short-term episodes of paroxysmal atrial fibrillation that are expected to end spontaneously within seven days. Some of the cases that are not evaluated with adequate examinations can be considered as included in the cryptogenic stroke group.^7^

Although the association of persistent atrial fibrillation with stroke is better known, paroxysmal atrial fibrillation is also blamed for the same risk of ischemic stroke as persistent atrial fibrillation and as a potential source of cryptogenic stroke.^8^

In a retrospective study on 3,480 patients with TIA or ischemic stroke, paroxysmal atrial fibrillation was found in 237 (19%) of the patients. In univariate analyses, the following were identified as important markers for paroxysmal atrial fibrillation: increasing age, female gender, previous ischemic stroke, myocardial infarction, other heart diseases, pathological troponin, embolic stroke and stroke in different arterial regions.^9^

Conditions that are known to be risk factors for both AF and stroke, such as age, male gender, hypertension, diabetes mellitus, valvular heart disease, heart failure, coronary heart disease, chronic kidney disease, inflammatory disorders, sleep apnea and tobacco use, have been shown to be responsible for the association between AF and stroke.^10^ In our study, no significant associations between presence of PAF and any factors other than advanced age were found.

About a quarter of strokes are recurrent.^11^ In our study, no significant relationship was found between the presence of PAF and the number of recurrent strokes. It is important to investigate the etiological factors in ischemic stroke and to arrange appropriate treatment, because this reduces the risk of recurrence of stroke. Since the presence of atrial fibrillation requires anticoagulant treatment, its detection is of particular importance. In the presence of atrial fibrillation, anticoagulation is the main treatment method for limiting systemic complications.^12^

In fact, the risk of stroke, which varies between 0 and 18% per year according to individuals’ clinical situation and risk profile, is not equally distributed among patients with atrial fibrillation. For this reason, it is important to evaluate thromboembolic risk in a personalized manner. A variety of scoring systems can be used for individualized patient selection. The CHA2DS2-VASc score is one of the most widely used scoring systems and is an effective method for considering the risk of stroke and the rate of anticoagulant benefit in patients with atrial fibrillation.^12^ Guidelines have recommended that anticoagulant therapy should be started if patients present nonvalvular AF with a CHA2DS2-VASc score of 2 or more.^13^ Since all the patients included in our study received a score of 2 points only because of ischemic stroke and this score increased in the presence of other risk factors, patients with AF are considered to be the patient group that will benefit from anticoagulant treatment. It is vital to reveal AF through Holter monitoring in these patients.

Since the 1960s, Holter monitoring has been the cornerstone for diagnosing suspected arrhythmias in patients of all ages. The length of the recording in the most commonly used monitoring systems is limited to 24-48 hours, while newer Holter monitors allow continuous electrocardiogram recording for two weeks.

Prolonging the ECG recording time will increase the diagnostic efficiency of Holter monitoring, especially for rare but recurrent rhythm disorders.^14^ However, long-term monitoring has disadvantages such as reduced patient compliance and increased cost. Ischemic stroke leads to the possibility of cognitive impairment, which may impair compliance among patients. Criteria for appropriate patient selection need to be developed.

Studies have shown that AF can be detected in approximately 10% of patients by extending the follow-up to 30 days, among patients who are examined with the diagnosis of ESUS. Moreover, AF can be detected in one fourth of patients by using continuous monitoring, for example, with implantable loop recording devices. However, attention has been drawn to the need for an algorithm for patient selection and for diagnosing progressive rhythm, considering that not all stroke patients can receive such intense monitoring.^15^

In a study in which 11,658 patients with stroke and atrial fibrillation were evaluated retrospectively between 1980 and 2014, the cardiac monitoring methods used were divided into four groups. In the first stage (in the emergency room), an admission electrocardiogram was performed. In stage 2 (in the hospital), serial ECG, continuous inpatient ECG monitoring, continuous inpatient cardiac telemetry and in-hospital Holter monitoring were performed. In stage 3 (first ambulatory period), ambulatory Holter was performed. In stage 4 (second ambulatory period), mobile cardiac telemetry, external loop recording and implantable loop recording were performed. The summary proportions of patients diagnosed with post-stroke atrial fibrillation were 7.7% in stage 1, 5.1% in stage 2, 10.7% in stage 3 and 16.9% (13.0-21.2) in stage 4. The overall yield of atrial fibrillation detection after all the stages of sequential cardiac monitoring was 23.7%.^16^

In our study, 33 (25.4) of the 130 patients had PAF attacks during the first Holter. Only 44 (45.4%) of the 97 patients whose participation in the second stage was planned were actually included in the study, and four (9.1%) of these 44 patients had PAF. Although the positivity rate decreased, the important point is that PAF was diagnosed as a result of investigative Holter among patients who had previously been examined. However, in order to gain statistical significance, this needs to be evaluated with a larger population.

Since Holter monitoring at our facility is limited to 24 hours, we aimed to evaluate the advantages and disadvantages of extending this period through repeated Holter recordings. We predicted that patients could become dependent on someone else due to their stroke, and that this could reduce compliance with appointment dates. In fact, 67 patients (51.5%) in our study had a score of 3 or more on mRs, and this was considered to be the dependent group. However, the major factor that reduced participation in the control Holter was the COVID-19 pandemic. It was not possible for this group of advanced age with chronic diseases to adapt to hospital controls during the pandemic period. The fact that PAF was only detected in the second recording, in four of the 44 patients who could be included in the control Holter, even though they had been scanned through 24-hour recording previously, draws attention to the insufficiency of 24-hour recording. The low participation in the control Holter suggests that it would be more appropriate to complete the examinations on this patient group, in which about half of these individuals were dependent on someone else to perform their daily activities, during hospitalization.

Another issue that we want to draw attention to in this study is the possibility of overlooking PAF if it is not evaluated through further examination, in the presence of other risk factors. Most of the chronic diseases that are considered to be risk factors for stroke, such as essential hypertension and congestive heart failure, are also closely related to atrial fibrillation. Therefore, their presence may play a role in the etiology of stroke, but is not sufficient to rule out the risk of PAF.

In our study, simultaneous PAF was detected in four of the 13 patients with symptomatic carotid stenosis detected through CT angiography and MRI angiography. This patient group was considered to present large-artery atherosclerosis according to the TOAST classification. Although the recommended Holter monitoring time for cases of cryptogenic stroke and ESUS was extended to 72 hours in the updated guidelines,^6^ the possibility that stroke might be multifactorial was ignored and no recommendations were made for these patients.

## CONCLUSION

Detecting the presence of PAF by scanning patients who did not show AF on ECG, through Holter ECG examination, is valuable in terms of changing the course of the treatment. It should be kept in mind that the possibility of accompanying PAF cannot be ruled out in the presence of other factors that pose a risk of stroke.
